# Synthesis and Biological Activity of Ultrashort Antimicrobial Peptides Bearing a Non‐Coded Amino Acid

**DOI:** 10.1002/psc.70021

**Published:** 2025-04-14

**Authors:** Cristina Peggion, Andrea Schivo, Martina Rotondo, Simona Oancea, Lucia‐Florina Popovici, Teodora Călin, Anna Mkrtchyan, Ashot Saghyan, Liana Hayriyan, Emma Khachatryan, Fernando Formaggio, Barbara Biondi

**Affiliations:** ^1^ Department of Chemical Sciences University of Padova Padova Italy; ^2^ Institute of Biomolecular Chemistry Padova Italy; ^3^ Department of Biology University of Napoli Naples Italy; ^4^ Department of Agricultural Sciences and Food Engineering “Lucian Blaga” University of Sibiu Sibiu Romania; ^5^ Laboratory of Diagnostic and Investigation, Directorate of Public Health Sibiu Romania; ^6^ Scientific and Production Center “Armbiotechnology” of NAS RA Yerevan Armenia; ^7^ Institute of Pharmacy Yerevan State University Yerevan Armenia

## Abstract

Antimicrobial resistance represents a significant global health threat, prompting the exploration of alternative therapeutic strategies. Antimicrobial peptides (AMPs) and lipopeptides are promising candidates due to their unique ability to disrupt bacterial cell membranes through mechanisms distinct from conventional antibiotics. These peptides are typically enhanced by motifs involving cationic amino acids, positive charge, and aromatic residues. Additionally, the conjugation of acyl chains to the N‐terminus of AMPs has been shown to improve their antimicrobial activity and selectivity. However, the susceptibility of peptides to enzymatic degradation presents a major limitation. To address this, we investigated the incorporation of non‐coded amino acids (NCAAs) to enhance peptide stability. Specifically, we synthesized the NCAA 2‐amino‐3‐(1*H*‐imidazol‐1‐yl)propanoic acid [His*], producing both enantiomers with high yield and optical purity. We then designed various analogs of ultra‐short AMPs by inserting His* at specific positions, evaluating their antimicrobial properties with different acyl chain lengths (C16 and C12) at the N‐terminus and the C‐terminus. We were able to identify a very promising candidate for applications (**P8**) characterized by resistance to proteolysis and enhanced biological effectiveness.

## Graphical abstract

Ultra‐short antimicrobial peptides containing acyl groups at the N‐terminus and the NCAA His* were prepared. The conformational analysis demonstrated that the peptides exhibiting the highest degree of structure were also those with the greatest antimicrobial activity. A promising candidate (**P8**) resistant to proteolysis with enhanced biological activity was identified.

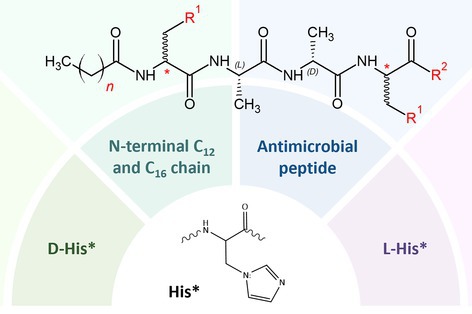

## 1 Introduction

Antimicrobial resistance is a major global health concern. Antimicrobial peptides (AMPs) and lipopeptides offer a promising alternative to conventional drugs in the fight against bacterial infections due to their ability to disrupt cell membranes, causing damage that is difficult to repair. They also act through mechanisms distinct from those of conventional antibiotics, which typically target specific cellular components. It is widely accepted that the antimicrobial activity of these peptides is enhanced by motifs that include cationic amino acids, a net positive charge, and aromatic residues [1]. Furthermore, as reported by Makovitzki et al., the conjugation of acyl chains to the N‐terminus of ultra‐short AMPs enhances their activity and selectivity against a broad range of microorganisms [2, 3]. This is because the aliphatic chain helps balance the short peptide moiety, aiding in the permeation and disruption of bacterial membranes, similar to the mechanism observed in many AMPs.

Examples of short AMPs that lack an aliphatic chain have also been reported in the literature [4]. Specifically, peptides rich in tryptophan, arginine or lysine demonstrated to be capable of interacting with negatively charged bacterial membranes. Commonly, these charged amino acids are alternated to hydrophobic residues forming amphipathic patterns [5]. For instance, the designed peptide MP196 (RWRWRW‐NH_2_), an amphipathic hexapeptide, targets the bacterial cytoplasmic membrane and inhibits cellular respiration and cell wall synthesis [6]. Additionally, other short peptides are able to form aggregates thus disrupting bacterial membranes [7].

A major drawback of using peptides as therapeutic agents is their susceptibility to degradation by the body's enzymatic systems. One potential solution is the introduction of non‐coded amino acids (NCAAs), which could enhance resistance to proteolysis.

**FIGURE 1:**
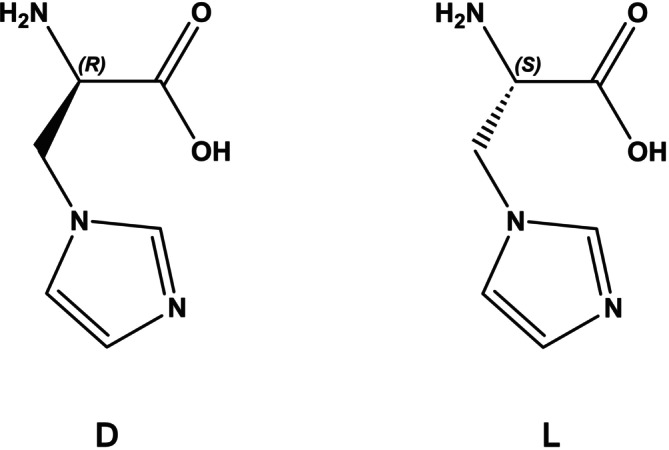
Structure of the NCAA 2‐amino‐3‐(1*H*‐imidazol‐1‐yl)propanoic acid (D‐ and L‐ enantiomers).

In our study, we focused on the His‐like, non‐coded amino acid (NCAA) 2‐amino‐3‐(1*H*‐imidazol‐1‐yl)propanoic acid [His*], synthesizing both enantiomers with high yield and optical purity (Figure 1) [8]. Starting with known ultra‐short AMPs containing a lipid moiety at the N‐terminus [2, 9, 10], we designed and synthesized various analogs by inserting His* at Positions 1 and 4. We considered the impact of both the L‐ and D‐enantiomers on the biological properties.

As acyl group at the N‐terminus, we inserted both a palmitoyl (C16) and a lauryl (C12) aliphatic chain to determine whether its length influences biological activity. Similarly, we investigated the effect of inserting the lipid chain at the C‐terminus, adding 11‐aminoundecanoic acid (Aun) at position 5 in the peptide sequence (Table 1).

**TABLE 1:** List of synthesized peptides.

	Peptide sequences
**P0**	Palm‐His‐Ala‐D‐Ala‐His‐NH_2_
**P1**	Palm‐L‐His*‐Ala‐D‐Ala‐L‐His*‐NH_2_
**P2**	Palm‐D‐His*‐Ala‐D‐Ala‐D‐His*‐NH_2_
**P3**	Ac‐His‐Ala‐D‐Ala‐His‐Aun‐NH_2_
**P4**	Ac‐L‐His*‐Ala‐D‐Ala‐L‐His*‐Aun‐NH_2_
**P5**	Ac‐D‐His*‐Ala‐D‐Ala‐D‐His*‐Aun‐NH_2_
**P6**	Laur‐His‐Ala‐D‐Ala‐His‐NH_2_
**P7**	Laur‐L‐His*‐Ala‐D‐Ala‐L‐His*‐NH_2_
**P8**	Laur‐D‐His*‐Ala‐D‐Ala‐D‐His*‐NH_2_

The final aim of this work was to assess the role of His*on both biological activity and resistance to proteolysis of the peptides.

## 2 Material and Methods

### 2.1 Peptide Synthesis

The solid‐phase peptide synthesis of the ultra‐short peptide analogs was carried out on the Rink Amide MBHA resin with a loading of 0.65 mmol/g, using standard Fmoc chemistry protocols [11, 12]. Deprotection of the Fmoc group was performed using a 20% piperidine solution in *N*,*N*‐dimethylformamide (2 × 10 min). For the activation of the carboxylic groups, a mixture in a three‐fold molar excess of amino acid, *N*‐*tert*‐butyl‐*N′*‐ethyl carbodiimide [13] (TBEC) and Oxyma Pure [14, 15] was used. Reaction time for each coupling was 50 min. On‐resin N‐terminus acylation was achieved pre‐activating the carboxylic acid (C16 or C12) in the presence of TBEC/Oxyma Pure. Fmoc‐Aun‐OH is commercially available (Iris Biotech GmbH, Marktredwitz, Germany) and was inserted using the same protocol as the other Fmoc‐amino acids.

Each peptide was cleaved from the resin using a mixture of trifluoroacetic acid (TFA), triisopropylhydrosilane (TIS), and water in a 95:2.5:2.5 ratio. The filtrates were collected and concentrated under a flow of nitrogen, and the crude peptide was precipitated by the addition of diethyl ether. The crude peptides were purified by flash chromatography on an Isolera Prime chromatographer (Biotage, Uppsala, Sweden) using a SNAP Cartridge KP‐C18‐HS 12 g or by preparative RP‐HPLC on a Phenomenex C18 column (22.1 mm × 250 mm, 10 μm, 300 Å) using an Akta Pure GE Healthcare (Little Chalfont, U.K.) LC system equipped with an ultraviolet detector (flow rate of 15 mL/min) and a binary elution system: A, H_2_O; B, CH_3_CN/H_2_O [9:1 (v/v)]; gradient from 25% to 55% B in 30 min.

The purified fractions were characterized by analytical HPLC–MS on a Phenomenex Kinetex XB‐C18 column (4.6 mm × 100 mm, 3.5 μm, 100 Å) with an Agilent Technologies 1260 Infinity II HPLC system and a 6130 quadrupole LC/MS instrument and by NMR spectroscopy (Supporting Information). All compounds were  >  95% pure.

### 2.2 Circular Dichroism (CD)

Electronic CD curves in the amide region (190–260 nm) were obtained on a Jasco (Tokyo, Japan) model J‐1500 spectropolarimeter. A fused quartz cell of 0.02 cm pathlength (Hellma, Mühlheim, Germany) was used to record spectra in phosphate buffer pH 7.4 (PB), and aqueous sodium dodecyl sulfate (SDS, 30 mM). Spectra were collected at room temperature with peptide concentrations of 1 mM. After baseline subtraction, the values recorded were transformed into [θ]_T_, the total molar ellipticity (deg × cm^2^ × dmol^−1^) by means of the JASCO software Spectra Manager.

### 2.3 Antimicrobial Tests

The antibacterial activity was tested on Gram‐positive ( *Staphylococcus aureus* ATCC 25923, *Streptococcus pyogenes* ATCC 19615, *Listeria monocytogenes* ATCC 7644, *Bacillus subtilis* ATCC –33, *Enterococcus faecalis* ATCC 29212, *E. faecalis* ATCC 51299, and some clinical isolates, *Streptococcus mitis* , *Streptococcus oralis* , *S. pyogenes* Group A and *Streptococcus* Group G), Gram‐negative ( *Klebsiella aerogenes* ATCC 13048, *Salmonella enterica* ATCC 1307, *Escherichia coli* ATCC 25922, *Pseudomonas aeruginosa* ATCC 27853, *Acinetobacter baumannii* ATCC 19‐0‐, *Klebsiella pneumoniae* ATCC 13883, *Proteu mirabilis* ATCC 14153) bacterial strains, and *Saccharomyces cerevisiae* ATCC 9763, according to the standardized disk diffusion Kirby–Bauer method [12, 16].

Peptide solutions in DMSO were adsorbed into paper disks (Macherey‐Nagel 615, Germany) of 6 mm in diameter. Every disk was loaded with 100 μg of peptide. The bacteria inoculum was of 0.5 Mc Farland, corresponding to 1–2 x 10^8^ CFU/mL. The tests were carried out on Mueller–Hinton culture medium at pH 7.2–7.4 with incubation at 37°C for 20 h. Peptide activity was determined by measuring the diameters (in millimeters) of bacteria/fungi growth inhibition around the paper disks.

### 2.4 Hemolytic Activity

The hemolytic activity of peptides was determined on sheep red blood cells according to the method described by Yamada and Natori [17].The experiments were approved by the Ethics Committee for Scientific Research Involving Human Subjects and/or Animals, of the “Lucian Blaga” University of Sibiu (process number 21/2024).

A stock solution of each peptide was prepared in DMSO, such as to obtain 1000 μg/mL. Five dilutions were made from the stock solution, using the PBS buffer pH 7.4.

Briefly, a suspension of sheep erythrocytes previously washed with PBS pH 7.4 was incubated with peptide solution in DMSO at five different concentrations (500, 250, 125, 62.6, and 31.25 μg/mL) at 37°C for 2 h. After centrifugation at 2000 *g* for 5 min, the absorbance of the supernatant was measured at 576 nm. Control sample by incubating sheep erythrocytes with PBS pH 7.4 was used, without peptide. The total hemolysis (100%) was measured by incubation of the erythrocyte suspension with distilled water.

Percentage of hemolysis was determined using the equation:

$$\%\text{hemolysis}=100\times \frac{A_{\text{sample}}-A_{\text{control}}}{A_{100}-A_{\text{control}}}$$

where:

*A*_*sample*_ = absorbance at 576 nm of the test sample.

*A*_*contro*l_ = absorbance at 576 nm of the control.

*A*_100_ = absorbance at 576 nm in case of total lysis.

### 2.5 Peptide Stability in Serum

The peptides were dissolved in DMSO at a concentration of 5 mg/mL. In Eppendorf tubes, 1 mL of HEPES buffer (25 mM, pH = 7.6) was temperature equilibrated at 37°C before adding 250 μL of human serum and 20 μL of peptide solution. The reaction was monitored for 24 h. At fixed intervals, 100 μL of the solution was withdrawn and added with 200 μL of absolute ethanol. The sample was kept on ice for 15 min and then centrifuged at 16,000 *g* for 5 min. Finally, the supernatant solution was analyzed by HPLC or HPLC–MS. [18].

A peptide of similar length, not resistant to degradation in serum, was used as a positive test. The peptide sequence is as follows: Tyr‐Ser‐Ser‐Phe‐Leu.

To verify peptide stability in buffer solution, samples containing peptide solutions, buffer, and ethanol were also analyzed. All of the degradation experiments were carried out in triplicate.

## 3 Results and Discussion

We focused on the peptide sequence Pal‐X‐Ala‐D‐Ala‐X‐NH_2_, [2, 19] where X represents a His residue [9]. We aimed to take advantage of the ability of NCAAs to improve stability to proteolysis by replacing the His residue with His*, characterized by a bond between the β‐carbon and one of the two nitrogen atoms in the imidazole ring. To investigate also the role of chirality, we synthesized both enantiomers of His* through asymmetric synthesis, obtaining them in high yields, and optical purity [8, 20].

**FIGURE 2:**
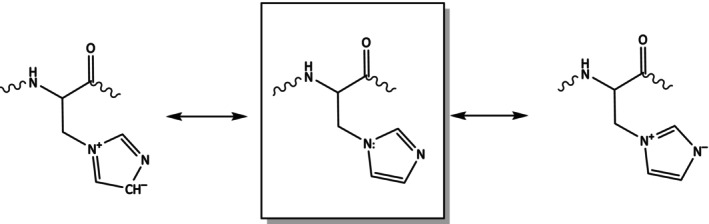
Resonance limit structures of His* residue.

His* preserves the aromaticity of the His residue, but it is a base slightly weaker, as 2‐alkylimidazoles have pka values of about 8.0, 1‐alkylimidazoles of about 7.2 [21]. The direct connection to nitrogen enables the identification of three distinct resonance structures (Figure 2).

The presence of all‐L amino acids in a peptide generally results in a highly hemolytic compound [22]. Therefore, with the aim of limiting the hemolysis, we synthesized analogs in which we introduced L‐ or D‐His* maintaining D‐Ala at position 3.

**FIGURE 3:**
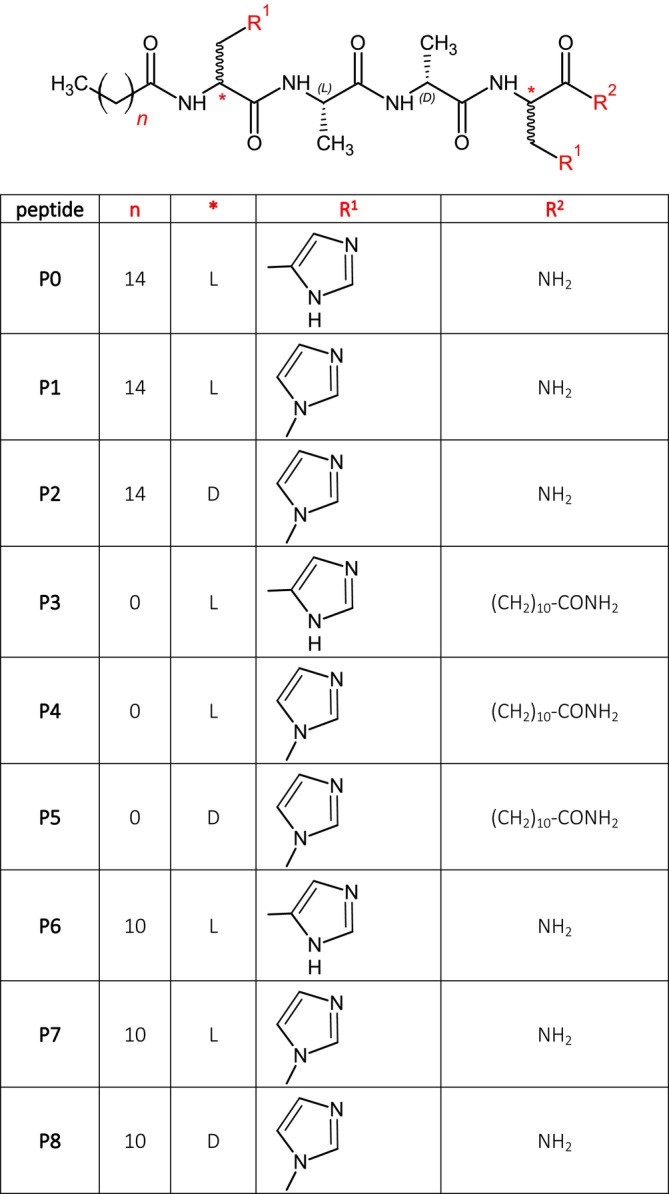
Molecular structure of the peptides synthesized.

Conjugation of acyl chains to the N‐terminus of ultra‐short antimicrobial peptides (AMPs) increases their activity and selectivity against different bacterial strains. Our study is based on ultrashort peptides proposed in the literature [2, 9], able to exert their activity only in the presence of a lipid chain. We expected a similar mechanism of action also when replacing His with His*. Additionally, here we evaluated the effect of chains of different length by synthesizing analogs in which we inserted a palmitoyl or lauryl moiety [23, 24]. To confirm the role of the acyl chain at the N‐terminus, we also shifted the lipid chain at the C‐terminus by introducing a residue of 11‐undecanoic acid and capping the N‐terminus with an acetyl group (Figure 3).

### 3.1 Peptide Synthesis and Characterization

All peptides were synthesized using the SPPS method using Rink Amide MBHA resin and Fmoc chemistry [25].

The desired products were obtained in good yields, with the purity of the crude peptides ranging from 70% to 96% (Table 2). The high purity of the crude peptides facilitated the purification step.

**TABLE 2:** Characterization data of the synthesized peptides.

Sample	R_t_ (min)^a^	Purity (%)	MW _calc_ ^b^	MW _exp_ ^b^
**P0**	20.0	97	671.9	671.4
**P1**	19.9	95	671.9	671.4
**P2**	19.8	95	671.9	671.4
**P3**	10.6	99	658.8	658.4
**P4**	10.6	99	658.8	658.3
**P5**	10.9	99	658.8	658.4
**P6**	16.0	96	615.8	615.3
**P7**	16.2	96	615.8	615.3
**P8**	16.3	95	615.8	615.3

^a^ Column Phenomenex Kinetex, 0.46 × 10cm, 100 Å, 3.5 μ; 5%–95%B over 30 min.

^b^ MW_calc_ and MW_exp_ are average masses.

### 3.2 Conformational Analysis

A CD analysis of the peptides in PB 10 mM at physiological pH and in the presence of SDS was performed in order to rationalize the different antimicrobial activities observed for our new compounds. The analysis of compounds with lauryl chains revealed discrepancies between the two experimental conditions investigated. **P6** showed a CD spectrum in PB that cannot be attributed to any known secondary structure, and a similar behavior was observed for this peptide in SDS. For the His*‐containing peptides **P7** and **P8**, partial structuring is already observed in PB with a spectrum shape reminiscent of a β structure. The intensity of the bands at 195 and 210 nm increased from PB to SDS, indicating an enhancement in the structural organization of the peptides.

**FIGURE 4:**
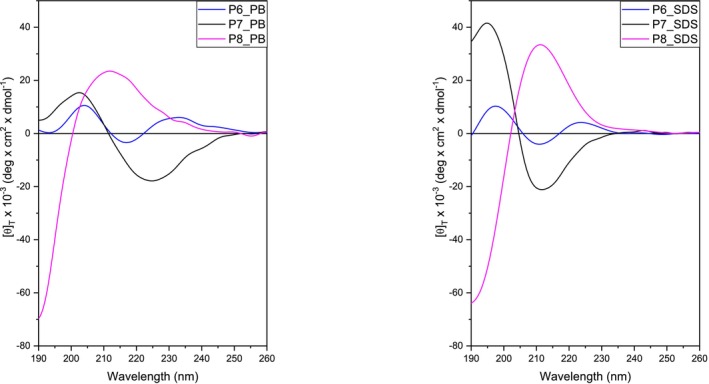
CD spectra in phosphate PB solution, pH 7.4 (left) and SDS solution (right) of selected peptides (**P6**, **P7**, and **P8**).

**P7** and **P8** contain His* residues of opposite configuration, resulting in dichroic bands with opposite signs in **P8** compared to **P7**, accordingly to the different D‐ and L‐ amino acid content in the two sequences (Figure 4).

In PB solution no signal was observed for the palmitoylated compounds (**P0**, **P1**, and **P2**), due to a poor solubility in water. In the presence of SDS the compounds bearing the palmitic moiety showed a similar behavior to that observed in those with the lauric chain (data not shown).

### 3.3 Antimicrobial Activity, Hemolysis, and Stability in Serum

The primary aim of this study was to assess the role of His*, a mimic of the natural amino acid His, on the antimicrobial activity of ultra‐short peptides derived from Pal‐X‐Ala‐D‐Ala‐X [2]. The synthesized peptides were tested against various bacterial strains, both Gram‐positive and Gram‐negative, as well as against *S. cerevisiae* .

The parent peptide (P0) is selectively active against *Salmonella enterica* ATCC 13076. When L‐His is replaced by L‐His* (P1) the activity is lost. However, when D‐His* (P2) replaces L‐His, the spectrum of activity is expanded to include several Gram‐positive strains, such as *Streptococcus pyogenes* ATCC 19615 and some clinical isolates.

When the lipidic chain is shifted from the N‐ to the C‐terminus no activity is observed for the parent peptide analog (**P3**) as well as for the analogs incorporating His* (**P4**, **P5**). Thus, the N‐terminal lipidic chain plays a pivotal role in the antibacterial activity of these ultra‐short peptides.

It was previously observed, in a naturally‐occurring class of lipopeptides, that a C12 chain has superior permeation ability of phospholipid double layers as compared to a C16 chain [26]. For this reason, we synthesized also three analogs (**P6**, **P7**, and **P8**) with a shorter carbon string at the N‐terminus. Interestingly, not only is the antimicrobial activity lost upon C‐lipidation recovered, but it is largely increased for the His* analogs **P7** and **P8**.

In both cases, a broad spectrum of activity was observed (Table 3).

**TABLE 3:** Antimicrobial activity against Gram‐negative bacteria, Gram‐positive bacteria, and *Saccharomyces cerevisiae* after 20 h from peptide incubation at 37°C*.* Activity was determined by measuring the diameters (in mm) of bacteria/fungi growth inhibition around the paper disks.

	Gram‐positive bacteria									Fungi	Gram‐negative bacteria					
**Peptide**	***B. Sub*. ATCC 6633**	***S. aur*. ATCC 25923**	***E. faec*. ATCC 29212**	***S. pyog*. ATCC 19615**	***S.mitis* (clinical isolate)**	***S. oralis* (clinical isolate)**	***S. pyog*. Gr. A (clinical solate)**	***Strept*. Gr. G (clinical isolate)**	***E.faec*. ATCC 51299**	***S. cerev*. ATCC 9763**	***K. aer*. ATCC 13048**	***S. ent*. ATCC 13076**	***E. coli* ATCC 25922**	***P. aer*. ATCC 27853**	***A. baum*. ATCC 19606**	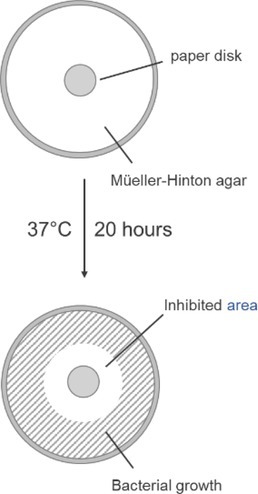
**P0**	—	—	—	—	—	—	—	—	—	—	—	10	—	—	—	
**P1**	—	—	—	—	—	—	—	—	—	—	—	—	—	—	—	
**P2**	—	—	—	10	8	8	8	—	—	—	—	8	—	—	—	
**P3**	—	—	—	—	—	—	—	—	—	—	—	—	—	—	—	
**P4**	—	—	—	—	—	—	—	—	—	—	—	—	—	—	—	
**P5**	—	—	—	—	—	—	—	—	—	—	—	—	—	—	—	
**P6**	—	—	—	—	—	—	—	—	—	—	—	9	—	—	—	
**P7**	11	9	12	—	—	—	—	—	—	12	10	8	9	8	—	
**P8**	12	12	9	17	10	—	12	—	—	14	9	—	7	8	—	

Particularly interesting results were obtained when these peptides were tested against *S. cerevisiae* .

*S. cerevisiae* is a yeast species that plays a dual role as a vital contributor to the fermentation industry and a key model organism in scientific research [27]. Although typically non‐pathogenic, there has been a growing frequency of *S. cerevisiae* isolations from various human organs. This rise is fueled by emerging reports of infections caused by this previously considered benign species [28]. While the exact frequency of severe infections, such as fungemia caused by *S. cerevisiae* , remains unclear, it is estimated that such cases may account for 0.1%–3.6% of all bloodstream fungal infections. The activity shown by the peptides **P7** and **P8** against *S. cerevisiae* is 12and 14 mm, respectively. These particularly high values pose them as interesting candidates as antifungal agents against *S. cerevisiae* .

**FIGURE 5:**
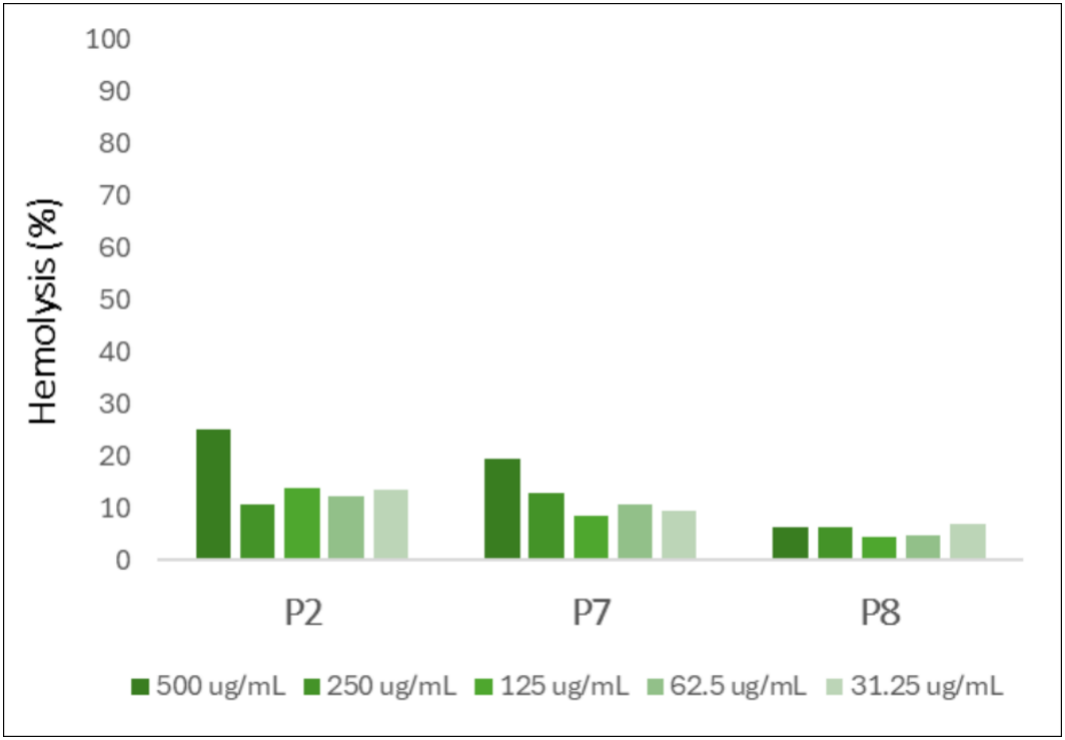
Hemolytic activity of the active peptides at five different concentrations, on sheep red blood cells.

The active peptides were tested for hemolytic activity on sheep red blood cells at five different concentrations. All the compounds analyzed exhibited moderate hemolysis, even at higher concentrations (Figure 5). Among the tested samples, **P8** emerged as the most promising candidate, showing a wide spectrum of activity and very low hemolysis.

**FIGURE 6:**
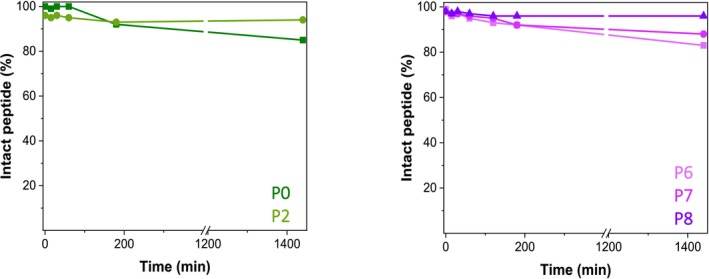
Percentage of intact peptide detected at defined time points over a period of 24 h.

All active peptides and the reference compound were tested for their stability against proteolytic degradation in serum, showing that approximately 80% of the peptides remained intact after 24 h. However, the introduction of His* (both isomers) contributed to greater stability over the 24‐h analysis period. The two best candidates in terms of serum stability are **P2** and **P8**, both containing the D‐His* residue (Figure 6) [29]. To further validate our findings, we also tested an analog of Leu‐enkephalin under the same conditions, a peptide known to be susceptible to serum degradation [30]. This compound was completely degraded within the first 30 min.

## 4 Conclusions

In this study, we focused on ultra‐short‐chain AMPs containing an acyl group at the N‐terminus. To enhance antimicrobial activity, we substituted the histidine residues in the peptide sequence with a NCAA, specifically using both enantiomers of His*. The conformational analysis demonstrated that the peptides exhibiting the highest degree of structure were also those demonstrating the greatest antimicrobial activity. This finding suggests that the presence of a secondary structure is an essential prerequisite for the mechanism of action. We also investigated the impact of the acyl chain length on antimicrobial efficacy, testing both C16 and C12 chains. The introduced substitutions led to improved antibacterial activity. Notably, the incorporation of D‐His* and the concurrent replacement of palmitic acid with lauric acid resulted in a peptide with broad‐spectrum activity, effective against both Gram‐positive and Gram‐negative bacteria, as well as *S. cerevisiae* . Hemolysis assays on sheep red blood cells showed that all active peptides exhibited low hemolytic activity. Additionally, these substitutions enhanced serum stability making **P8** the most promising candidate for future applications.

## Conflicts of Interest

The authors declare no conflicts of interest.

## Supporting information


**Figure S1** 1 H‐NMR spectrum of peptide P8 in DMSO, d6 solution (c = 1.5 mM, T = 25°C).
**Table S1.** 1 H Chemical shift assignment table for the peptide P8 in DMSO solution, c = 1.5 mM, T = 25°C.
**Figure S2.** HPLC profile corresponds to the stability in serum for peptide P8.

## Data Availability

The data that support the findings of this study are available on request from the corresponding author. The data are not publicly available due to privacy or ethical restrictions.
