# Supplementary material for: Synthesis and Biological Activity of Ultrashort Antimicrobial Peptides Bearing a Non‐Coded Amino Acid
Source: J Pept Sci. 2025 Apr 14;31(5):e70021. doi: 10.1002/psc.70021 (PMC11997541; doi:10.1002/psc.70021)
Supplement: Supplementary file 1 — Figure S1 1 H‐NMR spectrum of peptide P8 in DMSO, d6 solution (c = 1.5 mM, T = 25°C). Table S1. 1 H Chemical shift assignment table for the peptide P8 in DMSO solution, c = 1.5 mM, T = 25°C. Figure S2. HPLC profile corresponds to the stability in serum for peptide P8. [file PSC-31-e70021-s001.pdf]

SUPPORTING INFORMATION

Synthesis and biological activity of ultrashort antimicrobial peptides bearing a non-coded amino acid.

Cristina Peggion[1,2], Andrea Schivo[1], Martina Rotondo[2,3], Simona Oancea[4], Lucia-Florina Popovici[4], Teodora Călin[5], Anna Mkrtchyan[6,7], Ashot Saghyan[6,7], Liana Hayriyan[6,7], Emma Khachatryan[7], Fernando Formaggio[1,2], Barbara Biondi[2*]*

[1]Department of Chemical Sciences, University of Padova, 35131 Padova, Italy; [2]Institute of Biomolecular Chemistry, Padova Unit, CNR, 35131 Padova, Italy; [3]Department of Biology, University of Napoli, 80126 Napoli, Italy; [4]Department of Agricultural Sciences and Food Engineering, "Lucian Blaga" University of Sibiu, 550024 Sibiu, Romania; [5]Laboratory of Diagnostic and Investigation, Directorate of Public Health, 550178 Sibiu, Romania; [6]Scientific and Production Center "Armbiotechnology" of NAS RA, 0056 Yerevan, Armenia; [7] Institute of Pharmacy, Yerevan State University, 0025 Yerevan, Armenia

1. NMR characterization

As an example of the characterization of the synthesized peptides, we report the characterization for peptide **P8**:

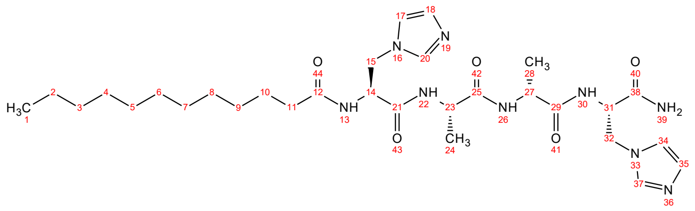

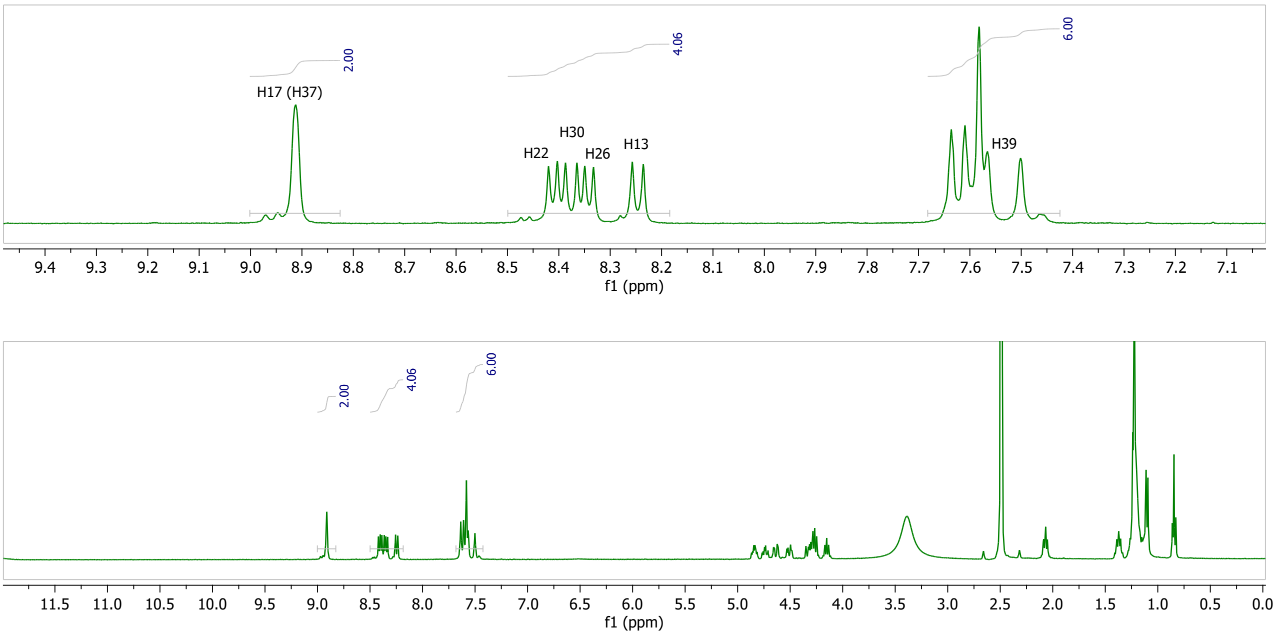

Figure S1. ^{1}H-NMR spectrum of peptide **P8** in DMSO, d6 solution (c= 1.5 mM, T=25°C).

Table S1. ^{1}H *Chemical shift* assignment table for the peptide **P8** in DMSO solution, c=1.5 mM, T=25°C.

Residue	NH (ppm)	α (ppm)	β (ppm)	Other (ppm)
Lau^0	-	-	-	H2 2.07
D-His*^{1}	8.25	4.84	4.51, 4.28	H2 8.92, H4 7.59
Ala^2	8.42	4.28	1.23	
D-Ala^3	8.35	4.16	1.11	
D-His*^{4}	8.38	4.76	4.65, 4.31	H2 8.92, H4 7.59
-NH_2				7.56, 7.50

2. *In serum stability*

As an example, we report the stability of peptide **P8** in the presence of human serum, monitored by HPLC–MS over 24 hrs.

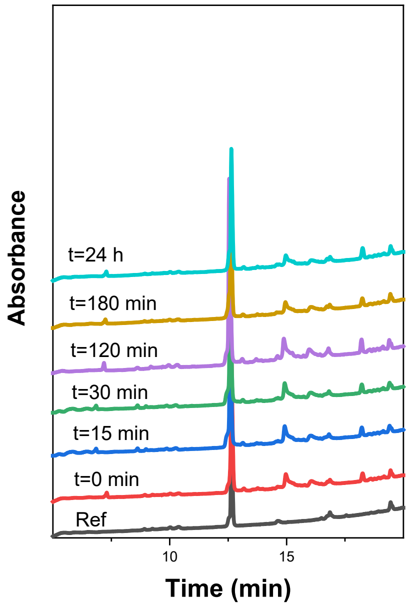

Figure S2. HPLC profile corresponding to the stability in serum for peptide **P8**.
